# Post-Graduate Study

**Published:** 1886-10

**Authors:** J. D. Moody

**Affiliations:** Mendota, Ill.


### THE DENTAL REGISTER.

VOL. XL.] OCTOBER, 1886. [No. 10.

### COMMUNICATIONS.

### Post-Graduate Study.

BY J. D. MOODY, D.D.S., MENDOTA, ILL.

"A college training is an excellent thing: but, after all, the better part of a man's education is that which he gives himself."

JAMES RUSSELL LOWELL.

I propose making this sentiment the key note of my paper. On two former occasions I have presented the subject of dental education,* and my intention is to take up at this time only one phase of this question, that of post-graduate study; to show its need, to outline a scheme of study, and to devise a plan for carrying it out. I believe that there is not a gentleman present who does not realize that some further elaboration of our educational scheme is, to say the least, desirable. Very many there are who think it imperative. To those who care nothing for a true scientific culture, and to whom the proper insertion of a beautiful filling or of an artistic denture is the *ultima thule* of dental ambition, this paper will have no interest. But to those who are longing for something better either in themselves or in the general profession, something which "will foster in us that spirit of broad and liberal thinking which is the essence of true scientific culture," to them I offer these suggestions as a partial solution of the question.

A physician is reported as having recently said that the practice of medicine in the near future is to be largely hygienic and sanitary, rather than one of drug prescribing. This may be

*Ohio State Journal of Dental Science, July, 1883. President's Address before the Central Illinois Dental Society, October, 1885.

rather a rosy view of the case, and yet it contains some truth; and while in all probability we will never get beyond the necessity of tooth filling, the time is surely coming when the dental surgeon will be assigned the lower seat and the dental doctor will be given the higher one. But before this time comes there must come a change in our educational methods. In a late number of the *Medical Record* there is an editorial on "Practicality in Medicine," in which the writer warns the profession against going to the extreme in making theory secondary to the practical, and uses the following language: "In our anxiety about the superstructure are we not getting careless about the foundations?" May not this apply as well to ourselves? Do we as dentists possess the foundation principles upon which to build a truly scientific superstructure? They are not to be found in the curriculum of any college. Even did they exist there, the usual two years course would be too short a time for their proper study. Three years would not give time enough. The mind of the average matriculate is not sufficiently matured to carry on such work. A young man destined for the ministry passes through the high school, then four years of college, and finally three years of seminary work. Can we be contented with anything less? As I shall show farther on, the list of subjects upon which every intelligent dentist should be fairly well informed, is far greater than any present college curriculum, and greater than under present circumstances it is possible to embrace in college work. A man cannot well remain in school long enough to gain that knowledge upon which to build great scientific laws, and it is folly to expect the student to pursue them beyond college life without some other incentive than the mere acquisition of knowledge. Of course there are men here and there, who can by sheer force of will accomplish this, but our aim should be to raise the profession as a whole to this high standard. How then are these foundation principles to be acquired? It must be by a life of study. A man can hardly begin serious work before the age of forty. He is then just in his prime, and, if the proper foundations have been laid, he is in a position to begin original work and render valuable service to his profession and to the world.

Are we, as dentists, making the most of our privileges? It seems to me that we are not; for, in glancing over the files of dental journals for ten years past, I was struck with the fact that so few new names have come to the front. You could almost count them on the fingers of your hands. Perhaps our profession stands as well as others in this regard, but I am sure this is not as it should be. In this country there are from fifteen to twenty thousand dentists. We can just as well be a generation of dental scientists as a generation of dental shopkeepers; but this will depend upon whether we lay the proper foundations. But about these foundations. Let me quote the opening paragraph of a paper read by Dr. Williams before the First District Dental Society of New York. He says: "I am sure you will rightly apprehend me when I say that the daily routine of details which goes to make up the practice of our specialty is not calculated to foster in us that spirit of broad and liberal thinking which is the essence of true scientific culture, and that it is therefore good for us to occasionally go back to a consideration of those *first principles, which are the foundations of all specialties.*"† The italics are mine. And this paragraph from Lionel Beale. "The observer who aims at studying the remarkable and highly interesting phenomena of germination, growth, and multiplication of the bioplasm of cells or elementary parts, in the tissues and organs of man in health and disease, will find it advantageous first to investigate those processes in the simplest living beings, where they occur under conditions less complex. * * * * The observer will learn many most important facts by watching the germination of the common mildew, and studying the different appearances of the plant when developed under different circumstances. * * * * The mode of origin and multiplication of a bacterium and the growth of a spongiole of a plant may appear to be questions far indeed removed from the province of medical inquiry, and yet, we shall find that by such investigations only can we hope to determine the nature of some phenomena, the true explanation of which lies at the very root of a knowledge of

†Cosmos, March, 1885, page 129

the real nature of disease. * * * * Let not the student of medicine, therefore, conclude that the multiplication and growth of the lower forms of animal and vegetable life are not in his province. *There is, indeed, scarcely a department of natural knowledge which does not bear more or less directly upon medicine.*"‡ Again the italics are mine. If this is true of the student of medicine, how much more is it true of the student of dentistry. Now what are these "first principles" to which Dr. Williams alluded?

Taking up his article, "Molecular Structure and Force with Reference to Nutrition," it will be seen that to read it understandingly it will be necessary for us to have a fair knowledge of of the theory of molecular dynamics, also of chemistry, inorganic, and the more complex organic, and of chemical physics. Truly that is getting down to first principles. And how about the statement of Lionel Beale? Just what shall be the scope of these studies of ours; in what department of natural knowledge?" I answer, in every thing that in any way bears upon the life history of organized matter.

Let us take a look through the library, and see whether this statement is too sweeping. I select a book here and there indiscriminately. First I take up some volumes of the "Independent Practitioner," containing articles on micro-organisms, by Dr. Miller. These theories and facts are of the utmost value to the dentist, but to thoroughly understand them we must be acquainted with all the teachings of microscopy and of bacteriology, and this latter must be based upon a previous knowledge of vegetable morphology and physiology and cellular metabolism, and this latter again upon molecular physics. Also a knowledge of chemistry and of chemical physics will be needed, and of animal anatomy and physiology as well. The little work on "Formation of Poisons," by Dr. Black, will require an equal range of knowledge on the part of the reader. Take up Foster's Physiology, or turn to the article on Physiology by Foster in the "Encyclopædia Britannica." To read these intelligently all of the above quoted studies must be thoroughly in mind, and in addition will be

‡The Microscope in Medicine, London, 4th ed., pages 135-6.

needed a knowledge of the lower forms of vegetable and animal life and of electricity. Here is a volume of the "Dental Register" for 1885. I turn to an article by Dr. Frank Abbott, on "Microscopical Studies upon Absorption of the Roots of Temporary Teeth." On attempting to read it, it will be found that we will need microscopical experience of no mean order, indeed of the very best, as also a knowledge of chemistry, cell metabolism, histology, and anatomy. Next I pick up a little pamphlet published by Dr. Barrett, of Buffalo, on "Nervous Force, Its Origin and Physiology." This is a subject with which we have much to do, and to have a good understanding of this article we must be at home in molecular physics, chemistry, anatomy, physiology, and histology, Take down the last volume of "The Reference Handbook of Medical Sciences," turn to the article "Endothelium," and see how our previous teachings have been at fault, based as they were upon erroneous deductions drawn from imperfect embryological investigations. With the development of the dental tissues, at least, we ought to be familiar. Dr. Atkinson has well said, "Direct interrogation of nature through embryological investigation to get at physiological changes, and examination of specimens under the microscope of tissues affected by diseased action, form the only possible way to arrive at a discriminative ability that will at all meet the demands of a high ambition to know."

Look through the files of the "Cosmos," or other dental journals, or of any medical journal, and notice the articles on nutrition, embryology, development, metallurgy, chemistry, etc., etc. It will require a high order of mental culture to be able to read them with ease and profit. Do you not see that Lionel Beale's statement, previously quoted, "There is indeed, scarcely a department of natural knowledge which does not bear more or less directly upon medicine," etc., is not too broad in its claim?

On every commencement day the advice is given to the graduates to keep apace with the current literature of their profession. It is good advice, but can be only partially followed out, for, as a rule, the graduates do not have the preliminary knowledge to enable them to grasp in detail a large part of it.

I believe that on an average, graduates are about twenty-five years old. At forty the man is in his prime; his intellectual faculties and his physical being are at their best. Could the spare moments of these intervening fifteen years be devoted to systematic study, they would fit the man at forty or forty-five to grasp the great problems confronting the profession, with an intelligent energy that would surely be productive of great results. The ripened years from forty to sixty-five, devoted to research, would lift the dental surgeon to the rank of a dental scientist. According to this plan, college life would be but a preparation of the ground for foundation building. From twenty-five to forty, the foundations would be in process of laying, and the superstructure erected in the afternoon of life.

But just what lines of study should be taken up in this work? To get at the more advanced thought in this direction, I addressed letters to several of the most widely known educators in the dental profession outside of our own state, asking them to outline briefly a plan of post-graduate study, "such as would be required to make a thoroughly scientific dentist." The burden of each reply was, more study. I quote from some of them to let you see what others are thinking about this matter. Dr. Barrett says: "There are many of the medical branches that are closely connected with the practice of dentistry. A graduate from a dental college should comprehend them, but his knowledge must be obtained by after study, as they are not in the dental curriculum." He enumerates advanced work in microscopy and histology, practical and experimental physiology. He further says: "In natural physics no one should neglect the study of the Forces. The Conservation and Correlation of Force should be carefully studied. Then the student is ready to take up Tyndall's Floating Matter in the Air, and Magnin's Bacteriology, without a study of which, one is not prepared to comprehend general physics." Dr. Wm. H. Atkinson, whose suggestions always carry great weight, writes me, (in addition to the sentence previously quoted,) the following, to which I desire to call especial attention. "I know of no dental college, and for that matter, of no medical college, that so instructs its matriculates as to make them possessors of even the alphabet of the molecular changes through which elemental substances pass in the production and maintenance of functioning bodies liable to the manifold activity, known as weakness and disease." Also the following sentence: "It is almost daily experience that the present demand of really earnest young men is for that which cannot be found without very extensive reading of text books and journals, and a long series of observations in practical studies, whereby they may become masters of diagnosis, prognosis, and prescription.

Dr. Frank Abbott, Dean of the New York College of Dentistry, who knows from experience how difficult it is to raise the standard of dental education, fears he may be considered extravagant in only demanding a thorough knowledge of anatomy and surgical anatomy of the head and face, the microscopical study of the teeth, their surroundings, and the pulp in health and disease, embryology, certainly as far as the development of the teeth are concerned, etc.

Dr. Taft, who, as teacher, editor, and author, is so well qualified to speak authoritatively, says, "the ordinary graduate usually has only a foundation upon which to build; most of the studies should be pursued much further than is required for graduation, and some branches that are not required at all should be taken up and studied. * * * * Gynecology, also should be more thoroughly studied; also camparative odontology. A far more extensive study of oral pathology and surgery should be required." And then he makes a suggestion in regard to advanced study, which I will only mention, as it is foreign to the thought I desire to develop; and yet I think it a very important one indeed, and worthy of careful consideration by thinking men. His idea is to arrange an advanced course, embracing four years of study and work. Those entering upon it would have it in view from the beginning; their work would be more thorough than that of the student competing for the D.D.S. Then when this has been fully accomplished, he would recognize it by conferring the degree of *Master of Dental Surgery.* This plan is worthy of a paper itself. But I recognize the difficulty of inducing any large number to enter upon this extended and expensive course of study. So long as one can graduate in two years and stand on the same legal footing with others, there will be a difficulty in securing students for the advanced course. But further than this there are at present hundreds in the profession who feel the need of just such work, but to whom it would be impossible to close up business for four years to enter upon such study. It is to this large class that my plan would be especially applicable.

But about the special studies. Out of my own experience in study and from long thinking on the subject, I have arranged the following scheme of studies. I would have them follow each other in about the order named, and preferably pursuing only one study at a time.

Physics, molecular.
mechanical movements.
Electricity, magnetism.
Chemistry, inorganic.
organic.
chemical physics.
Metallurgy.
Microscopy, technical.
Zoology, invertebrate, morphology.
Botany, systematic.
Anatomy, human.
comparative.
Embryology.
Odontology, comparative.
Histology, vegetable.
animal.
Physiology, vegetable.
animal.
chemical.
Bacteriology.
Pathology, general.
dental.

You will notice that I have not included in this list special disease or remedies, and general knowledge has not been considered at all. Further you will notice that each study follows logically upon the one which precedes it. I do not expect that any one would master these subjects in the allotted fifteen years. I would only expect a working knowledge of these to be obtained in that time. The best use to make of a library is not to sit down and try to master its contents, but to find out what information it contains, and where and how to obtain that information when wanted for use.

At forty or forty-five I should expect a man to have a good working knowledge of these subjects, and in all probability to have developed an inclination in some one direction of study, and then, and only then, would he be in a position to attempt real work or to have an intelligent understanding of such work when presented to him.

I do not see how any one study in this scheme can be left out, but I want to call especial attention to organic chemistry, as of vital importance to us, and yet hardly touched upon in college work; the same can also be said of chemical physiology. Bacteriology, too, which underlies the very foundations of dental pathology, has not received the attention it merits, and without radical changes in our methods of teaching cannot be properly taught in our schools.

I have only included in this list those branches, a knowledge of which it will be necessary for us to obtain, in order that we may have anything like an idea of the basal principles upon which our profession rests, and without which we cannot hope to grasp the meaning of, much less formulate, great scientific laws. A man may fill a tooth or treat an abscess without having acquired all this information, but without it his treatment is largely empirical. When the mass of the profession come to lay these solid foundations, a long stride will have been taken towards lifting our practice out of the empirical into the scientific.

Again, if we have the high ambition to make our profession a truly scientific one, we should do everything in our power to make it attractive to cultured minds. We can, if we will, gather the educated young men about us. It is within our power to make the name "dentist" an honored one. But to do this we must make our work appeal to the intellectual rather than to the sordid side of man's nature.

And again, in this day, when the tendency is to subdivide every branch of knowledge into specialties, the most work, and the best work is done by the man who, after having laid the proper foundations, devotes himself to one of these subdivisions. It will be necessary for anyone who hopes to make a decided mark in his calling to first lay the broad foundation which I have indicated.

Now comes the question about which the greatest diversity of opinion may arise, namely, how shall this course of study be carried out? First, the plan suggested to me by Dr. Taft could be adopted, and the outline of study I presented, or any similar one, be carried out. This, however, requires an attendance at school of at least four years, with its consequent expenses. The higher degree of Master of Dental Surgery would of course be quite an incentive to undertake this course. Secondly, the busy man, who could not give up practice to attend school, and yet has a real desire to know, by using this outline of study as a guide to a simple course of reading, could in a few years acquire a creditable fund of information.

I would not repress individuality in this work. A man's work must be part and parcel of himself, the outgrowth of his own mind, if he would have it make an impression upon the world. Yet there must be a certain uniformity in plan and methods among those who are working towards a common end.

The want of some plan in study, the knowing just what to take up, and in what order to make the most of it, has been felt and expressed many times. I have felt it myself. Some can formulate such a plan themselves to the very best advantage. The majority cannot. To meet some such want as this, has been my aim in presenting this tabulated course of reading. I am confident that for this purpose it will leave little to be desired. The individuality will have room for play when the foundation principles here laid down will have come to be put to practical test.

Lastly, the plan, which, all things considered, I think would be productive of the most good, is that of a Post-Graduate Correspondence School. Educational authorities throughout the country are coming to recognize the value to the busy man of this method of teaching. It has been put to a practical test in a score of places. Institutions of learning, scientific societies, and other bodies are using this means of instruction with increasing satisfaction. Under this system the members of the school do not leave their homes. They engage in all their usual vocations, and thus are not at expense of time or money. The instructors in each department should be thoroughly capable men, appointed to their position by the directors of the institution. It would be necessary to prepare text books, for it is a lamentable fact that we have not to-day a properly prepared dental text book. They must be accurate, abreast with the latest thought, and moderate in price. An exhaustive treatise is not a proper text book. Something after the plan of those used in Huxley's School of Biology, at South Kensington, would be needed. Such books should be prepared under the authority of some inter-collegiate organization. The pupil's work would be guided by correspondence with the tutor. Written examinations at monthly intervals, and yearly personal examinations probably should be held.

Only one, or at most two branches, should be carried on at a time, and the student should be unlimited as to time in which to do the work. This work should be thorough, and it is possible to make it so, and when accomplished it should be recognized by a degree meaning something. It should mean more than Doctor or Master of Dental Surgery. I would suggest D.D.Sc., standing for Doctor of Dental Science, Instead of calling this institution a School of Dental Surgery, I would call it an Institute or an Academy of Dental Science.

But we should organize such an institution? It should be left neither to personal nor to any college ambition to take up and so risk its standing. It could best be carried out through some national organization, such as the Association of College Faculties, the Association of Dental Examiners, or by a body of directors, constituted for this purpose by some such organization.

I expect the objection will be made that the busy practitioner has not time for such work. This objection has been made and met time and again. "Where there is a will there is a way."

"What man has done, man can do again." We owe it to our profession, to our fellow men, to ourselves, that we make the most of our privileges, and live not for self alone. Allow me in in this connection to quote the words of one who has had much to do with educational methods, who has come in contact with men in every station of life, and who has made a special study of this method of education. He says: "There are thousands of full-grown men * * * who are at their best intellectually, and who, with some leisure and much longing, believe they could do more than read. They want to study; to study in downright earnest; to develop mental power; to cultivate taste; to increase knowledge, to make use of it by tongue and pen and life. * * * They believe they could think and grow, speak and write. They are willing and eager to try. Out of minutes they could construct college terms. They have will enough, heart enough, brain enough to go on, to go through; and all this while the every-day life continues, with its duty for this hour and for that. They believe that into the closely woven texture of every-day home and business life, there may be drawn threads of scarlet, crimson, blue, and gold, until their homespun walls become radiant with form and color. * * * ."*

It is probably true that the full benefit of this scheme would most likely accrue to the young graduate who has long years before him, but any one with the desire, by going at it systematically and using the spare moments, can accomplish it all. I expect that some will consider me extravagant in these demands, will consider them chimerical, but I believe in them, for they are born of my convictions, and I verily believe that in some such line as this much of our future educational work is to be done.

---

*The Chautauqua Movement, Dr. J. H. Vincent, p. 178.

